# Mental health status among Burmese adolescent students living in boarding houses in Thailand: a cross-sectional study

**DOI:** 10.1186/1471-2458-13-337

**Published:** 2013-04-12

**Authors:** Takeshi Akiyama, Thar Win, Cynthia Maung, Paw Ray, Kayako Sakisaka, Aya Tanabe, Jun Kobayashi, Masamine Jimba

**Affiliations:** 1 Immunobiology Group, Department of Tropical Infectious Diseases, Center of Molecular Biosciences, Tropical Biosphere Research Center, University of the Ryukyus, Senbaru 1, Nishihara, Okinawa 903-0213, Japan; 2 The Mae Tao Clinic, 865 Moo 1, Intarakiri Road, Mae Sot, Tak 63110, Thailand; 3 Burmese Migrant Workers’ Education Committee, 644 Samasapkarm Road, Moo 2 Para Hta Pa Den, Mae Sot, Tak 63110, Thailand; 4 Department of Community and Global Health, Graduate School of Medicine, The University of Tokyo, 7-3-1 Hongo, Bunkyo-ku, Tokyo 113-0033, Japan; 5 Japan Association for Mae Tao Clinic, 1-1 Kaguragashi, Shinjuku-ku, Mail Box No. 52, Tokyo 162-0823, Japan; 6 Department of Global Health, School of Health Sciences, University of the Ryukyus, 207 Uehara, Nishihara, Okinawa 903-0215, Japan; 7 Bureau of International Medical Cooperation, National Center for Global Health and Medicine, 1-21-1 Toyama, Shinjuku-ku, Tokyo 162-8655, Japan

## Abstract

**Background:**

In Tak province of Thailand, a number of adolescent students who migrated from Burma have resided in the boarding houses of migrant schools. This study investigated mental health status and its relationship with perceived social support among such students.

**Methods:**

This cross-sectional study surveyed 428 students, aged 12–18 years, who lived in boarding houses. The Hopkins Symptom Checklist (HSCL)-37 A, Stressful Life Events (SLE) and Reactions of Adolescents to Traumatic Stress (RATS) questionnaires were used to assess participants’ mental health status and experience of traumatic events. The Medical Outcome Study (MOS) Social Support Survey Scale was used to measure their perceived level of social support. Descriptive analysis was conducted to examine the distribution of sociodemographic characteristics, trauma experiences, and mental health status. Further, multivariate linear regression analysis was used to examine the association between such characteristics and participants’ mental health status.

**Results:**

In total, 771 students were invited to participate in the study and 428 students chose to take part. Of these students, 304 completed the questionnaire. A large proportion (62.8%) indicated that both of their parents lived in Myanmar, while only 11.8% answered that both of their parents lived in Thailand. The mean total number of traumatic events experienced was 5.7 (standard deviation [SD] 2.9), mean total score on the HSCL-37A was 63.1 (SD 11.4), and mean total score on the RATS was 41.4 (SD 9.9). Multivariate linear regression analysis revealed that higher number of traumatic events was associated with more mental health problems.

**Conclusions:**

Many students residing in boarding houses suffered from poor mental health in Thailand’s Tak province. The number of traumatic experiences reported was higher than expected. Furthermore, these traumatic experiences were associated with poorer mental health status. Rather than making a generalized assumption on the mental health status of migrants or refugees, more detailed observation is necessary to elucidate the unique nature and vulnerabilities of this mobile population.

## Background

Thailand’s economic growth has attracted increasing numbers of migrant workers from neighboring countries [1]. Within the Thai society, migrants are among the most vulnerable groups [2,3], made up frequently of families with children taken to or born in Thailand [2]. Yet little research has yet been done on the mental health status of these children, and very little quantitative information is available to clarify the issue [2].

A large number – possibly half – of Burmese migrants in Thailand might be regarded as refugees, considering their backgrounds [4]. Many migrants from Myanmar have described experiences of violence, displacement due to conflict, forced relocation, conscription for labor, rape, taxation, and other harassment. Moreover, a lingering fear of being forced to return to one’s country of origin is a common experience in such populations [4-6].

A Thai Cabinet Resolution approved free education up to grade 12 for all children in Thailand [1]. However, it is difficult for Burmese migrants to attend Thai schools for a number of reasons including language, cost, fear of deportation, and curriculum [1,2,7]. In Tak province, thousands of Burmese migrant children attend migrant schools (officially defined as “learning centers” in the Thai education scheme) [7], which have been built by the local Burmese migrant community.

At the time of this study, there were 68 migrant schools and 28 boarding houses for their students in Tak. Local Burmese migrant schools and migrant organizations manage these boarding houses. The age of the residents of these boarding houses ranges widely, from infants to adolescents up to 18-year-old. The local migrant community and NGOs donate towards and supervise these Burmese migrant schools and boarding houses to protect students from exploitation.

According to the teachers, children and adolescents may live in these boarding houses for various reasons. Some students have lost their parents; others have been sent away from Myanmar by their parents. Aside from familial financial situations, poor living conditions in conflict zones, including the danger of landmines [8-10], might have prompted parents to send their children to boarding houses in Thailand. In the case of refugee adolescents, those who have experienced traumatic events and have been separated from their parents or primary caregivers are an important group that requires attention and support [11-17]. Such individuals must often cope with traumatic events, including loss of home, family and belongings, without the support of parents or caregivers [18,19]. The incidence of mental illnesses such as post-traumatic stress disorder (PTSD) and depression is relatively high among these adolescents [17-22].

Social support is often beneficial to psychological wellbeing [23-26]. Low levels of social support are an important predictor of mental health problems, including symptoms of PTSD [27]. Indeed, a qualitative study of unaccompanied refugee adolescents between the ages of 15 and 18 in Belgium suggested that the provision of social support enhanced their wellbeing [28]. At the time of this study, however, little was known about the characteristics and mental health status of Burmese adolescent students in Thai boarding houses, as few quantitative studies have been conducted to investigate social support in such adolescents.

### Objectives of the study

This study aimed firstly to elucidate the demographic characteristics and mental health status of Burmese adolescents residing in boarding houses in Thailand. Further, the study aimed to examine the factors related to mental health status among such students.

## Methods

### Ethical considerations

Written informed consent was obtained from all participants and their guardians prior to enrollment in the study. Further, all information collected in this study was kept anonymous to ensure the confidentiality of participants. None of the information could be used to identify any of the participants, and the collected data were used for research purposes only. The Research Ethics Committee of the University of Tokyo reviewed and approved the protocol of this study, under registration No. 2655. The Coordinating Team for Displaced Children’s Education Burma, which consists of local migrant organizations and coordinates the protection of the children, also approved the study protocol.

### Study design and study area

This cross-sectional study was conducted in Tak province, Thailand. Tak is located 426 km from Bangkok, near the Myanmar border. As of 2000, the population was 484,356 [29]. Tak has one of the highest rates of entry into Thailand for migrants from Myanmar. In a 2004 registration drive, the number of work permits issued to migrants from Myanmar in Tak totaled about 50,000 – the third highest number within all Thai provinces [1].

### Study participants

The target population of this study was Burmese adolescent students residing in migrant boarding houses in Tak province. Boarding house students aged from 12 to 18 years were selected through a convenience sampling method based on practical reasons, such as having been included in previous research using similar questionnaire types [20], the possibility of their being included in follow-up, the ability to provide them with treatment for any mental health problems, and their ability to understand how to fill out the questionnaires.

### Sampling procedures

The inclusion criteria for participant enrollment were as follows: (1) aged between 12 and 18 years, (2) attending school in grades 7 to 12, and (3) residing in a boarding house. Participants included students from different ethnic backgrounds including Burmese, Karen, and Mon. All students who took part in the study understood either Burmese or the Karen language, as these were the primary languages of instruction in the migrant schools. At the time of this study, there were a total of 28 boarding houses in Tak province.

One boarding house, which accommodated ethnic Shan students who were studying in a formal Thai school, was excluded from this study due to a divergence in the language of instruction compared with the other boarding houses, and the associated difficulties in coordinating its involvement. This study also excluded boarding houses that accommodated only students of lower ages and grades than the aforementioned criteria. As a result, only16 boarding houses were ultimately identified for inclusion in this study.

Overall, the total number of targeted students in the 16 boarding houses was 771. All 771 students were invited to participate in data collection, which was conducted in their school classrooms on Saturdays or Sundays. Of these recruited students, 428 (55.5 %) agreed to participate in the study and 304 completed the questionnaire in full.

### Measurement of demographic characteristics and mental health status

The demographic information collected in this study included each student’s age, grade, sex, years spent in Thailand, and length of time (in years) that they had resided in their boarding house. The study also asked the whereabouts of each of their parents, whether that was “in Thailand”, “in Burma”, in some “other country”, “dead”, or “unknown”.

To measure the symptoms of mental health problems among the student participants, this study employed the Hopkins Symptom Checklist-37 for adolescents (HSCL-37A) [30]. The HSCL-37A is self-administered and is a modified version of the well-known HSCL-25 [30]. The HSCL-37A assesses internalized and externalized problems such as anxiety and depression. It has 37 questions and uses a four-point Likert rating scale [31].

The Stressful Life Events (SLE) checklist [32] was used to assess the number and types of stressful events that had been experienced by the participants. The scores were calculated from 12 self-administered dichotomous (yes/no) questions and one open-ended question. The items cover both the direct experience of physical mistreatment and the witnessing of others’ trauma. [32].

The Reactions of Adolescents to Traumatic Stress questionnaire (RATS) was also used in this study. This scale assesses post-traumatic stress reactions defined by the DSM-IV [33,34]. The RATS is a self-administered questionnaire that consists of 22 items and uses a four-point Likert scale. The questions are designed to measure symptoms of intrusion, numbing/avoidance, and hyperarousal, specifically among adolescent refugees [35]. The HSCL-A 37, SLE checklist, and RATS have all been used broadly in national surveys in the Netherlands and Belgium, particularly in studies of adolescent refugees [30-32].

### Measurement of perceived social support

In this study, perceived social support among participants was measured with the Medical Outcomes Survey (MOS) Social Support Survey Scale [36,37]. This scale has been carefully developed from previous instruments, and is generally considered to be based on a sound theoretical framework [38].

The MOS Social Support Survey Scale is self-administered and consists of 19 items, wherein the respondent indicates their perceived level of currently available social support using a five-point Likert scale. For example, the items ask about the availability of “someone you can count on to listen to you when you need to talk”. Responses options for such items are “none of the time”, “a little of the time”, “some of the time”, “most of the time”, or “all of the time.” This scale consists of four subscales: emotional/informational support, tangible support, affectionate support, and positive social interaction.

The MOS Social Support Survey Scale is reproduced with permission from the RAND Corporation. RAND's permission to reproduce the survey is not an endorsement of the products, services, or other uses in which the survey appears or is applied. Although permission to translate the survey was granted by RAND, the translation itself was not approved or reviewed by RAND.

### Questionnaire development

Translators from the local migrant community translated all of the aforementioned questionnaire scales into the Burmese and Karen languages. A Karen version was also prepared because education is provided in the Karen language in several Karen schools, and some students would not be able to fully understand a Burmese language questionnaire.

The Burmese questionnaire was filled out by the Burmese adolescents, and by other minority groups such as the Shan and Mon, who are also educated in the Burmese language in the migrant schools. The Karen questionnaire, meanwhile, was used by Karen students who were educated in the Karen language. Different translators conducted back-translations of the questionnaires to ensure meaning equivalence.

Regarding the Cronbach's alpha coefficients of the HSCL-37A and RATS, values obtained for the Burmese versions were as follows: 0.88 on the HSCL 37-A scale, 0.78 on its anxiety subscale, 0.81 on its depression subscale, and 0.89 on the RATS; Meanwhile, Cronbach’s alpha for the Karen version were as follows: 0.88 on the HSCL-37A scale, 0.83 on its anxiety subscale, 0.80 on its depression subscale, and 0.83 on the RATS.

The subscales of the MOS Social Support Survey Scales had the following Cronbach’s alpha coefficients for the Burmese version: 0.87 on the emotional/informational support subscale, 0.85 on the tangible support subscale, 0.87 on the affectionate support subscale, and 0.82 on the positive social interaction subscale. Cronbach’s alpha values for the Karen versions of the MOS Social Support Survey subscales were relatively modest: 0.65 on the emotional/informational support subscale, 0.73 on the tangible support subscale, 0.74 on the affectionate support subscale, and 0.69 on the positive social interaction subscale.

### Data collection

Data collection was conducted between September and October 2009. All of the Burmese students residing in the boarding houses were invited to participate in the study, and the research assistants were recruited from local NGOs. A mental health professional trained the research assistants in data collection. The assistants then distributed a recruitment document written in Burmese or Karen, as appropriate. The participants were able to choose either language. Using this document, the assistants explained the purpose and nature (e.g., confidentiality) of the research and also gave the students a verbal explanation about it in Burmese or Karen. Students who agreed to participate were asked to sign informed consent forms written in the appropriate language. The assistants explained to the participants the voluntary nature of participation and that anyone could leave the study at any time or skip any questions they did not want to answer. Contact information for counseling services was also provided to participants so that they could seek confidential help if they felt uncomfortable or encountered any problems.

The research assistants explained the aims of the research to school principals and to the students’ guardians, who were also asked to sign informed consent forms. School principals had the option to exclude any students who might have had difficulty in participating due to an outstanding emotional or physical condition. Contact information for counseling services was provided to students and school principals alike so that they could call for support if they encountered any student with any emotional or behavioral problem.

Participants filled out the questionnaire anonymously while in the classroom. They could select the questionnaire in their language of preference (Burmese or Karen). Mental health professionals and medical staff were present at the survey sites, and a pair of research assistants in each classroom provided verbal guidance while the students were filling out the questionnaire. The participants were also monitored in case any of them required emotional or healthcare attention. The research assistants ensured that breaks and relaxation were taken in the intervals between different sections of the questionnaire to help relieve any tension felt by the participants. The questionnaire and accompanying procedures took about 3 hours to complete in total. Following completion of the questionnaire, relaxation exercises and lunch were provided to the participants.

Out of 428 participants, seven were excluded through failure to meet one or more of the inclusion criteria. A total of 304 participants submitted completed questionnaires. Demographic characteristics such as age and gender distribution did not differ significantly between those who had submitted completed questionnaires and those who did not. For example, there was no median age (16 years) difference between the participants who submitted incomplete questionnaires and those who submitted completed questionnaires. The ratio of males to females was about 1:1 in both groups of participants. In addition, although the researchers could not obtain complete information about the participants who did not participate in this study, the median age of the 647 students eligible for this study (out of a total of 771) was also 16 years.

### Data analysis

T-tests were used to determine the mean differences between male and female participants in the number of traumatic experiences reported from the SLE checklist, scores on the MOS social support survey scales, HSCL-37A total scores, anxiety subscale scores, depression subscale scores, and RATS scores.

Each completed questionnaire was assigned an ID number. The participant’s demographic characteristics, any traumatic events experienced, mental health status, and MOS score were described. Multivariate stepwise linear regression analysis was then conducted to predict the total score, the scores on the depression and anxiety subscales of the HSCL-37A, and the total RATS score.

For multivariate linear regression analysis used to predict mental health status, the following items were used as independent variables: the number of traumatic experiences reported, age, gender, years spent in boarding houses, years spent in Thailand, whereabouts of parents, language of the questionnaire (Burmese or Karen) selected by the participants, and perceived availability of social support from MOS. The scores from subscales, rather than the total score, were used as recommended by Sherbourne and Stewart [37]. Stepwise multivariate linear regression analysis was performed for all variables simultaneously.

SPSS version 12 for Windows (SPSS, Inc., Chicago, IL, USA) was used for statistical analysis. The significance level for all T-tests was set at 0.05. In multivariate linear regression analysis, it was set at 0.0125 by Bonferroni correction, because four independent variables were used for analysis (scores of HSCL-37A, Depression subscale, Anxiety subscale, and RATS).

## Results

### Demographic characteristics

Table 1 shows the demographic characteristics of the participants. The students’ median age was 16 years. Out of all 304 students who submitted completed questionnaires, 208 (68.4%) chose to complete the questionnaire in Burmese while 96 (31.6%) chose Karen. The majority (n = 222, 73%) of the participants had been residing in Thailand for more than 1 year, while 63 (20.7%) had been there for less than 6 months. The median duration of residence in Thailand was 3 years and the mean was 4.5 years. Among all participants, 191 (62.8%) had been residing in boarding houses for more than 1 year. The median number of years spent in boarding houses was 3 and the mean was 3.5.

**Table 1:** Demographic characteristics of participants

**Characteristics**	**Total (n = 304)**		
		**n**	**(%)**
Gender	Male	163	(53.6)
	Female	141	(46.4)
Age (years)	Median, Range	16	12–18
	Inter-quartile range	(25%: 15, 75%: 17)	
Language	Burmese	208	(68.4)
	Karen	96	(31.6)
Length of residence in	0–5 months	63	(20.7)
Thailand	6–11 months	19	(6.3)
	More than 1 year	222	(73.0)
	Median, Range of years	3	1–18
	Inter-quartile range of years	(25%: 2, 75%: 6)	
Length of residence in	0–5 months	92	(30.3)
boarding houses	6–11 months	21	(6.9)
	More than 1 year	191	(62.8)
	Median, Range of years	3	1–11
	Inter-quartile range of years	(25%: 2, 75%: 5)	
Whereabouts of parents	Both parents in Myanmar	191	(62.8)
	Both parents in Thailand	36	(11.8)
	Father and mother both dead and mother is in Myanmar	20	(6.6)
	Father in Thailand, Mother in Myanmar	9	(3.0)
	Father dead, Mother in Thailand	9	(3.0)
	Other	39	(12.8)

A large proportion (n = 191, 62.8%) of the participants indicated that both of their parents lived in Myanmar, while only 11.8% answered that both of their parents lived in Thailand. Overall, only 6 participants indicated that both their father and mother were dead.

### Traumatic experiences

Table 2 shows the various reported traumatic events and the number of participants who experienced those events, as reported in the SLE checklist. Two hundred and eleven (69.4%) of the participants had seen someone suffer physical mistreatment. A large proportion (n = 180, 59.2%) had also experienced a stressful event whereby they witnessed someone in great danger. The mean total number of traumatic events experienced by the participants was 5.7 (standard deviation [SD] 2.9).

**Table 2:** Distribution of traumatic events in LSE

**Traumatic events**	**Total (n = 304)**		**Male (n = 163)**		**Female (n = 141)**	
	**n**	**(%)**	**n**	**(%)**	**n**	**(%)**
Experienced important changes in family life	166	(54.6)	94	(57.7)	72	(51.1)
Was separated from family against will	96	(31.6)	51	(31.3)	45	(31.9)
Experienced the death of a loved one	118	(38.8)	63	(38.7)	55	(39.0)
Had a life-threatening medical problem	106	(34.9)	57	(35.0)	49	(34.8)
Experienced a serious accident	111	(36.5)	62	(38.0)	49	(34.8)
Experienced a disaster	117	(38.5)	61	(37.4)	56	(39.7)
Experienced war or armed conflict	177	(58.2)	89	(54.6)	88	(62.4)
Was physically mistreated	123	(40.5)	75	(46.0)	48	(34.0)
Saw someone else physically mistreated	211	(69.4)	118	(72.4)	93	(66.0)
Experienced sexual abuse	34	(11.2)	23	(14.1)	11	(7.8)
Experienced a stressful life event in which ‘I was in danger’	132	(43.4)	79	(48.5)	53	(37.6)
Experienced a stressful life event in which ‘someone else was in danger’	180	(59.2)	97	(59.5)	83	(58.9)
Other frightening event	152	(50.0)	82	(50.3)	70	(49.6)
Mean number of events (Standard deviation)	5.7	(2.9)	5.8	(2.8)	5.5	(3.0)

In addition, the traumatic events experienced between male and female participants are described in Table 2. The mean number of traumatic events experienced was 5.8 (SD 2.8) for the male participants and 5.5 (SD 3.0) for the female participants. However, the t-test results did not show this difference to be significantly different.

### Perceived social support

Table 3 shows the distribution of responses from participants on the MOS Social Support Survey Scale. In particular, the response option selected by the largest number of respondents (n = 61, 20.1%) was “all the time” for availability of “someone who shows you love and affection”. The mean total score from the MOS Social Support Survey Scale was 2.7 (SD 0.7; Table 3).

**Table 3:** Distribution of scores on the MOS Social Support Survey scale (n = 304)

				**None of the time**		**A little of the time**		**Some of the time**		**Most of the time**		**All of the time**	
	**Mean**	**Median**	**SD**	**n**	**(%)**	**n**	**(%)**	**n**	**(%)**	**n**	**(%)**	**n**	**(%)**
Total Score	2.68	2.66	0.68										
Emotional / informational support	2.48	2.38	0.71										
Someone you can count on to listen to you when you need to talk	2.66	3.00	1.01	28	(9.2)	115	(37.8)	115	(37.8)	23	(7.6)	23	(7.6)
Someone to give you information to help you understand a situation	2.56	2.00	0.95	32	(10.5)	123	(40.5)	111	(36.5)	23	(7.6)	15	(4.9)
Someone to give you good advice about a crisis	2.59	3.00	1.01	46	(15.1)	105	(34.5)	102	(33.6)	30	(9.9)	21	(6.9)
Someone to confide in or talk to about yourself or your problems	2.33	2.00	1.11	69	(22.7)	129	(42.4)	65	(21.4)	20	(6.6)	21	(6.9)
Someone whose advice you really want	2.61	2.00	1.12	48	(15.8)	105	(34.5)	93	(30.6)	34	(11.2)	24	(7.9)
Someone with whom to share your most private worries and fears	2.16	2.00	1.08	92	(30.3)	119	(39.1)	60	(19.7)	17	(5.6)	16	(5.3)
Someone to turn to for suggestions about how to deal with a personal problem	2.38	2.00	1.06	57	(18.8)	131	(43.1)	77	(25.3)	20	(6.6)	19	(6.3)
Someone who understands your problems	2.56	2.00	1.00	36	(11.8)	120	(39.5)	109	(35.9)	19	(6.3)	20	(6.6)
Tangible support	2.90	2.75	1.01										
Someone to help you if you were confined to bed	2.76	2.50	1.24	40	(13.2)	112	(36.8)	76	(25.0)	32	(10.5)	44	(14.5)
Someone to take you to the doctor if you need it	3.05	3.00	1.24	23	(7.6)	97	(31.9)	86	(28.3)	38	(12.5)	60	(19.7)
Someone to prepare your meals if you were unable to do it yourself	3.00	3.00	1.27	30	(9.9)	97	(31.9)	78	(25.7)	42	(13.8)	57	(18.8)
Someone to help with daily chores if you were sick	2.78	3.00	1.23	38	(12.5)	113	(37.2)	75	(24.7)	35	(11.5)	43	(14.1)
Affectionate support	3.03	3.00	1.01										
Someone who shows you love and affection	3.17	3.00	1.21	21	(6.9)	77	(25.3)	95	(31.3)	50	(16.4)	61	(20.1)
Someone to love and make you feel wanted	2.91	3.00	1.29	44	(14.5)	83	(27.3)	85	(28.0)	41	(13.5)	51	(16.8)
Someone who hugs you	3.02	3.00	1.24	29	(9.5)	89	(29.3)	89	(29.3)	42	(13.8)	55	(18.1)
Positive social inter action	2.63	2.67	0.94										
Someone to have a good time with	2.72	3.00	1.10	29	(9.5)	119	(39.1)	95	(31.3)	30	(9.9)	31	(10.2)
Someone to get together with for relaxation	2.47	2.00	1.14	66	(21.7)	97	(31.9)	97	(31.9)	20	(6.6)	24	(7.9)
Someone to do something enjoyable with	2.69	3.00	1.13	42	(13.8)	101	(33.2)	97	(31.9)	36	(11.8)	28	(9.2)
Additional item													
Someone to do things with to help you get your mind off things	2.48	2.00	1.14	64	(21.1)	102	(33.6)	90	(29.6)	25	(8.2)	23	(7.6)

Among the subscales of the MOS Social Support Survey Scale, the mean score for emotional/informational support was 2.5 (SD 0.7), for tangible support was 2.9 (SD 1.0), for affectionate support was 3.0 (SD 1.0), and for positive social interaction was 2.6 (SD 0.9). The t-test results showed that mean scores on all subscales did not differ significantly between male and female participants.

### Mental health status

Distribution of scores from the HSCL-37A and RATS portions of the questionnaire are shown in Table 4, with higher scores indicating more mental health problems. The mean score for the HSCL-37A was 63.1 (SD 11.4). The mean scores for the HSCL-37A subscales, meanwhile, were as follows: 18.1 (SD 4.2) on the anxiety subscale, and 28.9 (SD 6.5) on the depression subscale. The mean total score for the RATS was 41.4 (SD 9.9).

**Table 4:** Mental health status

		**Mean**	**SD**	**95% CI**
Total (n = 304)	HSCL-37A total	63.1	11.4	61.9-64.4
	Anxiety	18.1	4.2	17.6-18.6
	Depression	28.9	6.5	28.2-29.6
	RATS total	41.4	9.9	40.3-42.5
Males (n = 163)	HSCL-37A total	61.6	10.6	60.0-63.3*
	Anxiety	16.9	3.7	16.3-17.5*
	Depression	28.3	6.3	27.3-29.3
	RATS total	40.7	9.6	39.2-42.2
Females (n = 141)	HSCL-37A total	64.9	12.0	62.9-66.9
	Anxiety	19.4	4.4	18.7-20.2
	Depression	29.6	6.6	28.5-30.7
	RATS total	42.2	10.2	40.5-43.9

* The difference in the mean between males and females was statistically significant (p < 0.05).

The distribution of scores from the HSCL-37A and RATS between males (n = 163) and females (n = 141) are also shown in Table 4. The mean HSCL-37A score was 61.6 (SD 10.6) for males, compared with 64.9 (SD 12.0) for female participants. The mean anxiety subscale score was 16.9 (SD 3.7) for male and 19.4 (4.4) for female participants. The mean depression subscale score was 28.3 (SD 6.3) for male and 29.6 (SD 6.6) for female participants. Finally, the mean RATS scores were 40.7 (SD 9.6) and 42.2 (SD 10.2), for males and females, respectively. The difference between males and females in the means on the HSCL-37A and on the anxiety subscale was statistically significant (Table 4).

### Perceived social support, mental health status, and sociodemographic characteristics

Table 5 shows the results of forced entry and stepwise multivariate linear regression analysis of the participants’ characteristics and mental health status. One finding from these final models of stepwise regression analysis was that female gender was associated with reporting higher scores on the HSCL-37A in both forced entry and stepwise models. The total number of traumatic events was identified as the strongest predictor in the subscales of the HSCL-37A and on RATS. However, the language of the questionnaire (Burmese or Karen) was significant for the HSCL-37A total scale score, anxiety, and depression. Age, whereabouts of the parents, and years spent in a boarding house did not appear to have a relationship with reported causes of distress in the total or subscale scores of the HSCL-37A or RATS after Bonferroni correction. Years spent in Thailand, however, appeared to be significant in predicting scores on anxiety subscale.

**Table 5:** Final model of stepwise linear regression analysis of the mental health status of participants

		**Forced entry**		**Step wise**							
				**Step 1**		**Step2**		**Step3**		**Step4**	
	**Predictor variable**	***Β***	**t**	***Β***	**t**	***Β***	**t**	***Β***	**t**	***Β***	**t**
HSCL-37A total	Age	0.02	0.39								
	Years in boarding house	0.01	0.14								
	Years in Thailand	−0.11	−1.95								
	Burmese language (Ref = Karen)	−0.22*	−3.97			−0.23*	−4.58	−0.24*	−4.89		
	Male gender (Ref = female)	−0.17*	−3.37					−0.19*	−3.70		
	Traumatic events	0.41*	8.17	0.41*	7.84	0.40*	7.90	0.41*	8.27		
	Social support										
	Emotional/ Informational support	0.03	0.40								
	Tangible support	−0.07	−1.20								
	Affectionate support	0.11	1.80								
	Positive social interaction	−0.05	−0.81								
R^2^	0.28			0.17		0.22		0.26			
Ajusted R^2^	0.25			0.17		0.22		0.25			
Anxiety	Age	−0.10	−1.96								
	Years in boarding house	−0.01	−0.22								
	Years in Thailand	−0.14*	−2.59							−0.15*	−3.18
	Burmese language (Ref = Karen)	0.22*	−4.22					−0.26*	−5.56	−0.23*	−4.67
	Male gender (Ref = female)	−0.31*	−6.63			−0.33*	−6.56	−0.34*	−7.20	−0.34*	−7.30
	Traumatic events	0.41*	8.78	0.40*	7.50	0.42*	8.39	0.41*	8.58	0.40*	8.66
	Social support										
	Emotional/ Informational support	0.04	0.55								
	Tangible support	−0.04	−0.68								
	Affectionate support	0.11	1.95								
	Positive social interaction	−0.08	−1.42								
R^2^	0.37			0.16		0.26		0.33		0.35	
Adjusted R^2^	0.35			0.15		0.26		0.32		0.34	
Depression	Age	0.11	1.98**								
	Years in boarding house	0.04	0.55								
	Years in Thailand	−0.12*	−1.94								
	Burmese language (Ref = Karen)	−0.19*	−3.39*			−0.19*	−3.72	−0.20*	−3.91		
	Male gender (Ref = female)	−0.13*	−2.55*					−0.13*	−2.59		
	Traumatic events	0.37*	7.17*	0.38*	7.07	0.37*	7.06	0.38*	7.27		
	Social support										
	Emotional/ Informational support	−0.04	−0.57								
	Tangible support	−0.05	−0.74								
	Affectionate support	0.13	1.96								
	Positive social interaction	−0.08	−1.24								
R^2^		0.23			0.14		0.18	0.20			
Adjusted R^2^		0.21			0.14		0.17	0.19			
RATS	Age	0.09	1.65								
	Years in boarding house	0.02	0.30								
	Years in Thailand	−0.13**	−2.17			−0.15**	−2.24	−0.12**	−2.28		
	Burmese language (Ref = Karen)	−0.07	−1.16								
	Male gender (Ref = female)	−0.10**	−2.01					−0.11**	−2.11		
	Traumatic events	0.45*	8.82	0.45*	8.77	0.45*	8.78	0.46*	8.94		
	Social support										
	Emotional/ Informational support	0.01	0.18								
	Tangible support	−0.05	−0.80								
	Affectionate support	0.14*	2.19								
	Positive social interaction	−0.09	−1.45								
R^2^		0.26		0.20		0.22		0.23			
Adjusted R^2^		0.23		0.20		0.21		0.22			

*: *p* < 0.0125, **: *p* < 0.05.

The perceived availability of social support score was not significant in predicting any of the dependent variables with Bonferroni correction (*p <* 0.0125). In the forced entry model, we found a relationship (β = 0.14) between affectionate support and higher RATS score, although the p-value was not significant (p = 0.03). Hence, the contribution of affectionate support does not appear as a variable in the stepwise regression.

## Discussion

Among the participants of this study, reporting traumatic events and female gender were associated with higher probability of mental health problems such as depression and anxiety. However, none of the subscales of the MOS scale was significantly associated with mental health problems after Bonferroni correction.

The participants in this study might have experienced a large number of traumatic events. The mean number of traumatic events was 5.7 across all surveyed students. A study from Belgium, also using SLE, revealed an average number of 3.6 traumatic events among adolescent migrants who arrived in the country (aged between 11 and 18 years) [19]. Like many other studies that have indicated an association between traumatic events and poor mental health status [39], there was a strong association between the number of adverse life events and mental health problems among the participants of this study.

Unexpectedly, a statistically significant association was not detected between perceived social support and mental health problems. Further studies covering more details such as the gender and source of support [40] might elucidate the roles of social support among adolescent students in boarding houses. Other studies involving Asian migrants or students have reported positive associations between social support and mental health status [41-44]. The participants of this study, in contrast, stayed in boarding houses and were thus separated from their families. Although this source of social support was not investigated in the present study, the participants’ main source of social support might thus be from peers, whereas the availability of family support would be limited. Notably, previous studies have suggested the important role of family support [45].

Furthermore, we found a relationship between affectionate support and higher RATS score, although the association was not significant after Bonferroni correction. However, reciprocity [46-49] and the specific source of the support [50,51] may be considered as additional factors affecting this issue. Understanding support and negative outcomes must take into account many dimensional pathways [50], not all of which could be addressed in the present study.

Overall, female participants reported higher levels of anxiety and depression than did their male counterparts. However, female gender alone is not adequate to explain the association with reporting poorer mental health status. Rather, the different factors associated with gender among the participants of this study would be expected to influence the results in this direction [52,53]. The notion of gender is socially constructed, encompassing culturally dictated conventions, roles, behaviors, and identities [54]. Gender and health status should thus be understood in the context of the manners in which people work, live, eat, and recreate [54]. In addition, the unique gender roles in Asian culture might influence the results in the translated scales [55]. Further, females might be more sensitive than men to their health status and more willing to report symptoms of distress [56]. Certain findings might thus be attributed to such gender differences in reporting depression and anxiety, and to an inherent gender bias in the measurement construct used in this study [57]. In other words, the effect of gender bias of the scales might partially explain the results observed [58,59].

Participants’ age was not a significant predictor variable in the stepwise multivariate regression analysis but weakly associated with the depression subscale in the forced entry model. However, few consistent results have hitherto been shown regarding the influence of age on mental health status among those affected by traumatic experiences [60]. Hence, further investigation is needed to elucidate this point.

The number of years spent in Thailand was a significant protective factor for mental health status. The whereabouts of parents and the number of years spent in boarding houses, on the other hand, were not. However, these factors should be analyzed in context and should account for further details such as the participant’s level of acculturation, adaptation, adjustment to their new country, and expansion of their social network within the community [61-63].

Among the participants of this study, the mean scores on the HSCL-37A and RATS scales were 63.1 and 41.4, respectively. Although it is difficult to draw comparisons due to the limited number of participants in this study, results from a mixed sample of 1,294 adolescent immigrants and refugees aged 11–18 years surveyed in a Belgian study indicated a mean total score of 56.9 for the HSCL-37A and 39.3 for the RATS [30,35]. This suggests a better overall mental health status than among the participants of this study. Notably, when the Belgian research sample was limited to only the 477 participants who were migrants, the mean score was 56.9 for the HSCL and 38.5 for the RATS.

This study has four primary limitations. First, the cross-sectional design could not assess the causal relationship between social support and the mental health status of the participants. A qualitative and longitudinal study would better serve to understand the process and effect of social support on the mental health status of students in boarding houses.

Second, data collected in this study did not cover detailed information such as the nature and role of the person(s) providing support to the students and building relationships with them [55,64]. The protective effect of social support on an individual’s well being is explained in two ways [65,66]. One is through a “buffering”-effect model of social support, which protects individuals from the influence of life stress. The other is through a main-effect model, by which social support directly benefits a person’s health status [66]. As information on the daily life stresses experienced by participants was not collected in this study, the buffering effect of social support could not be assessed.

Third, choosing the Burmese version of questionnaire was associated with greater frequency of mental health problems among students. Those participants who elected to fill out the Burmese version of the questionnaire also reported a poorer mental health status than did those who chose the Karen version. It is important to note that using a particular language would not be expected to associate directly with mental health status. The language itself is not a factor, but rather, as mentioned in the case of gender bias, it should be regarded as a proxy for different backgrounds and personal experiences.

Furthermore, those who chose the Burmese questionnaire included the Burmese adolescents and other minority groups such as the Shan and Mon. Further studies should be conducted considering potential difference across the ethnic communities [41]. Context validity of the questionnaire between Burmese and Karen versions might also be a source of bias.

Finally, although this study invited all 771 students in grades 7 through 12, the initial number of participants was only 428. The authors presumed that a major reason for the reduced participation was that the data collection was conducted only on Saturdays and Sundays, and that participation was voluntary, as a result of which many students would have elected to preserve their free time rather than participate. Furthermore, due to possible emotional distress that was associated with some questions, it was emphasized that participants could skip questions that they did not wish to answer. The questionnaire also contained a large number of items for the students to complete; thus some participants may not have been able to maintain consistent concentration throughout and could thus have overlooked items in the questionnaire. As a result, only 304 participants completed the questionnaires.

Despite these limitations, this study provides a valuable overview and insight into the mental health status of students in the boarding houses of Burmese migrant schools in Thailand. Mixed movement of refugees and migrants is a global phenomenon [67]. The refugee and migrant populations use the same route to the destination country and share similar risks such as human trafficking [67]. It is necessary to observe the nature and characteristics of the mobile population, rather than making generalized assumptions about illegal migration [67].

Psychiatric information about adolescents in low- and middle-income countries is generally sparse, but a troubling picture of depression and high suicide rates has been highlighted [68]. More comprehensive improvement and expansion of social services offered is necessary such as upgrading of mental health assessment tools, treatment in primary care, availability of medication, national mental health programs, and training of mental health care professionals [68]. At the same time, adolescents tend to underutilize mental health services due to stigma and other priorities in life [39]. Further studies are necessary to make mental health promotion more successful in low- and middle-income countries, particularly within such vulnerable populations [68,69].

## Conclusions

Experiencing a higher number of traumatic events was associated with having poorer mental health status among adolescents living in the boarding houses of Burmese migrant schools in Thailand. This result suggests that further studies are necessary to explore the mental health of adolescents who are subject to the strains of international migration and displacement.

## Competing interests

The authors declare that they have no competing interests.

## Authors’ contributions

TA conceived the study and developed the study design. KS helped in developing the study design and methodology. TW, CM, PR, and AT participated and helped in the data collection. JK and MJ reviewed and proofread the manuscript. All authors read and approved the final manuscript.

## Pre-publication history

The pre-publication history for this paper can be accessed here:

http://www.biomedcentral.com/1471-2458/13/337/prepub
